# Unique Presentation and Novel Surgical Approach to a Transcribriform Penetrating Head Injury Caused by a Nail Gun

**DOI:** 10.7759/cureus.25581

**Published:** 2022-06-01

**Authors:** Benjamin K Hendricks, Joseph D DiDomenico, Michael T Lawton, Andrew S Little

**Affiliations:** 1 Department of Neurosurgery, Barrow Neurological Institute, St. Joseph’s Hospital and Medical Center, Phoenix, USA

**Keywords:** endoscopic, nail-gun injury of head, penetrating head injury, pseudoaneurysm, transcribriform head injury

## Abstract

A penetrating head injury caused by a nail gun is an infrequent clinically diverse condition that varies in severity by the neurovascular structures involved. The authors present the case of a patient whose frontal lobe was pierced by a nail that entered via a transnasal transcribriform trajectory without causing vascular injury or intracranial hemorrhage; the man was unaware of the nail’s presence and presented with headache five days after the incident. The nail was extracted using a bifrontal craniotomy for direct visualization and for defect repair of the skull base combined with endoscopic endonasal extraction of the nail.

## Introduction

The most renowned case of survival after a penetrating head injury (PHI) is that of Phineas Gage, who had a tamping iron propelled through his left frontal lobe in 1848 [1]. Neurological outcomes after these injuries vary but can be surprisingly favorable, particularly after low-velocity PHIs [2]. Unlike high-velocity mechanisms, such as gunshots, low-velocity mechanisms translate to less kinetic energy and contribute to less surrounding shear injury [3,4]. The mechanism of injury, the presence and location of the foreign body, and the extent of penetration contribute to the long-term morbidity of patients with PHIs.

One type of low-velocity PHI is a nail-gun injury of the cranium. Since nail guns were introduced in the 1950s, they have become commonplace tools in home and work settings [5]. Nail-gun injuries have since become an increasingly frequent cause of emergency department visits [6]. However, the subset of nail-gun injuries that are intracranial account for fewer than 0.1% of all reported nail-gun injuries. Although intracranial nail-gun injuries have typically been characterized as the result of accidental or workplace-associated trauma [3,7,8], recent reports reflect an increase in intentional self-inflicted injuries [8-13].

We report a case of a penetrating nail-gun injury that has several notable features, including the benign transnasal trajectory of the nail into the frontal lobe, the delayed presentation of the patient for medical care, and the unique surgical management of the PHI. The patient consented to the publication of this case report and gave informed consent for surgery. Institutional review board approval is not required by our institution for reports involving fewer than three patients.

## Case presentation

**Figure 1:**
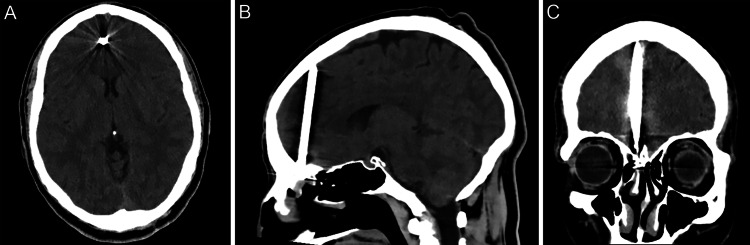
Preoperative computed tomography showing a transcribriform penetrating head injury caused by a nail gun. (A) Axial, (B) sagittal, and (C) coronal computed tomography showing an 8.3 × 0.5-cm nail penetrating the patient’s right cribriform plate and fovea ethmoidalis, projecting along the right mesial frontal lobe, and abutting the frontal inner table.* Used with permission from Barrow Neurological Institute, Phoenix, Arizona.*

**Figure 2:**
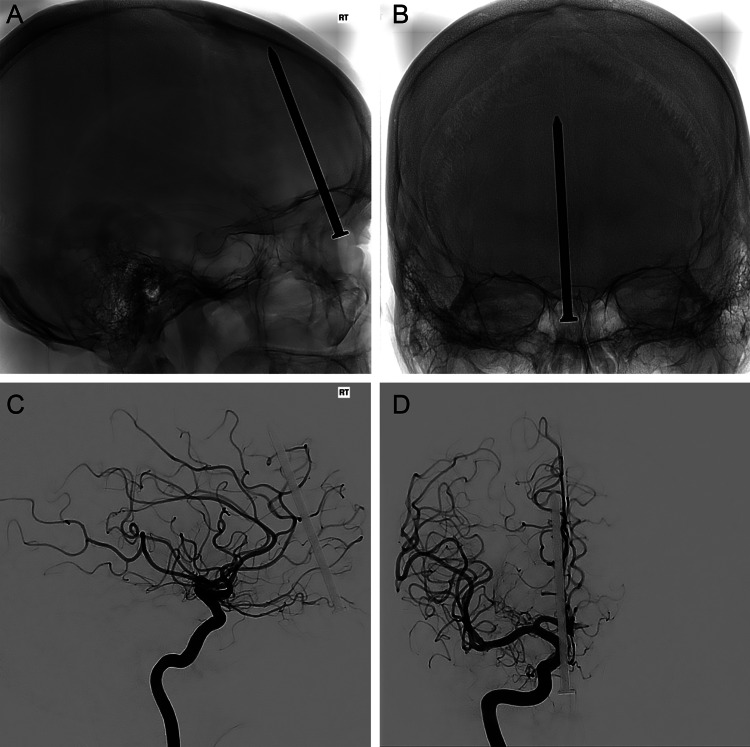
Preoperative radiographic and angiographic imaging showing the absence of vascular injury or intracranial hemorrhage. Preoperative imaging demonstrated no aneurysm, pseudoaneurysm, vascular injury, arteriovenous shunting, or active extravasation. Unsubtracted (A) lateral and (B) anteroposterior radiographs of the skull demonstrating the sagittal and coronal projection of the nail, respectively. Digitally subtracted (C) lateral and (D) posteroanterior angiography demonstrating a silhouette of the nail and no obvious vascular injury after right internal carotid artery contrast injection. *Used with permission from Barrow Neurological Institute, Phoenix, Arizona.*

A man in his forties presented with a persistent throbbing headache five days after sustaining an occult injury caused by a nail gun. He reported that the nail gun had discharged, propelling a nail that deflected off sheet metal and ricocheted into his face. He believed that the nail had struck the top of his mouth above his teeth. Physical examination revealed evidence of trauma to the sublabial region, suggestive of an entry point. The patient was neurologically intact and had no signs of cerebrospinal fluid (CSF) leak. Computed tomography of the head (Figure 1) demonstrated the nail projecting through the cribriform plate and entering the right frontal lobe immediately adjacent to the interhemispheric fissure. There was no sign of hemorrhage related to the PHI. Preoperative computed tomography angiography and digital subtraction angiography were both negative for vascular injury (Figure 2).

**Figure 3:**
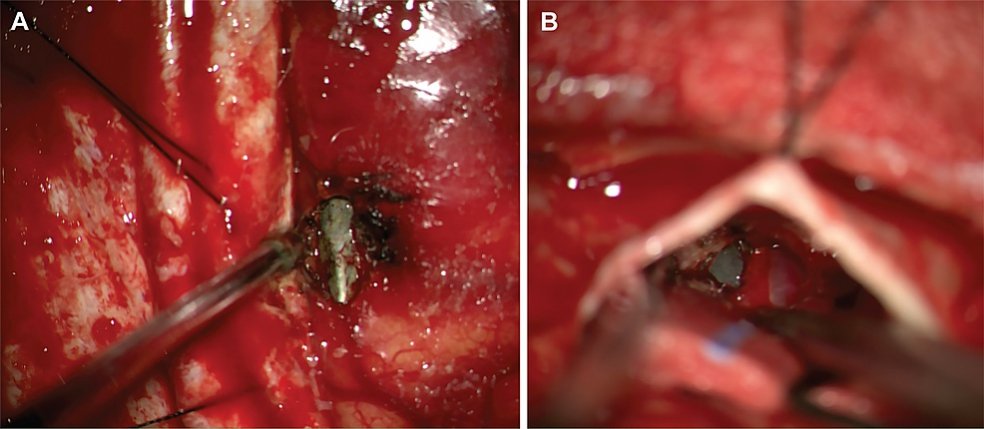
Intraoperative photographs showing the surgical removal of the nail. Intraoperative photographs demonstrate (A) the initial operative encounter with the nail, which was located within the right frontal parenchyma adjacent to the interhemispheric fissure, and (B) the nail traversing the cribriform plate and projecting into the right basal frontal parenchyma. *Used with permission from Barrow Neurological Institute, Phoenix, Arizona.*

An operation was planned to extract the nail endoscopically under direct intracranial visualization in case of bleeding. The patient was taken to the operating room for a bifrontal craniotomy. The dura mater over the right frontal lobe was opened, and the tip of the nail was encountered immediately adjacent to the interhemispheric fissure (Figure 3). A subfrontal approach was performed, and the body of the nail was located where it had penetrated the cribriform plate and dura and had entered the basal frontal lobe (Figure 3). Once the body and tip of the nail were completely visible, an endoscopic endonasal approach was performed to remove it. The nail was extracted by slowly pulling it from the endonasal cavity with Kelly curved forceps while pushing it from the intracranial cavity. No bleeding resulted from the nail extraction.

After removal of the nail, a complex defect repair of the skull base was performed to prevent CSF leak. Primary repair of the anterior fossa dura was performed using Gore-Tex suture (Gore Medical, W. L. Gore & Associates, Flagstaff, AZ). The bony defect was packed with a temporalis muscle plug covered by a small piece of DuraGen Secure matrix (Integra LifeSciences Corp., Plainsboro, NJ). The matrix was covered with Adherus dural sealant (HyperBranch Medical Technology, Inc., Durham, NC), and a pericranial flap was placed to overlay the dural defect.

Postoperatively, the patient remained at his neurological baseline. No CSF leak occurred postoperatively, and the patient was discharged home on postoperative day two after completing a short course of 1 g of vancomycin daily, 500 mg of metronidazole every eight hours, and 2 g of cefepime twice daily for two days. At the patient’s six-week postoperative follow-up visit, he remained neurologically intact, had no CSF leak, and had no signs of infection; computed tomography angiography did not demonstrate a delayed pseudoaneurysm.

## Discussion

The history of nail-gun PHIs is well described in the medical literature. Two review articles summarized 83 unique cases that were treated from 1959 to 2010 [14,15]. A PubMed search using the terms nail gun and penetrating head injury identified an additional 16 cases published from 2010 to 2017 [2-4,8,10-13,16-18]. Low-velocity penetrations, such as nail-gun PHIs, are considered less likely to produce focal neurological deficits than high-velocity penetrations. This tendency is the result of the lower kinetic energy of the projectile compared to that of a bullet. When this lower energy is transmitted to the parenchyma, it is less likely to result in a temporary cavitation effect. The temporary cavitation effect represents the radial displacement and shear of parenchyma adjacent to the path of a projectile, and the extent of this effect greatly increases morbidity subsequent to the injury [19].

Our case similarly exemplifies the remarkably benign clinical course that a nail-gun PHI can take, considering the lack of morbidity despite a five-day delay to treatment, the astoundingly benign trajectory of the nail, the well-tolerated operative interventions, and the exemplary postoperative course. Such a considerable delay in treatment has not previously been reported in the peer-reviewed literature, particularly with the patient unaware of the presence of the retained nail. These factors, in addition to the novel use of the endoscope to guide transnasal nail extraction, make this case a unique example of PHI.

Reports on surgical interventions for nail extraction are highly variable in the extent of exposure undertaken, ranging from closed traction to craniotomy for direct visualization [14,15]. Complication management can require more specialized surgical access if the patient has a vascular injury, CSF leak, or bone fragment displacement [3,12,16,18]. To our knowledge, no case report has yet documented the combination of an endoscopic endonasal extraction and transcranial direct visualization.

Given the risk of vascular injury from PHI, it is imperative to obtain preoperative vascular imaging, even in the absence of hemorrhage. Angiographic irregularities observed with these injuries include traumatic arterial dissection, stenosis, vasospasm, vessel penetration, and pseudoaneurysm. Pseudoaneurysm development is rare but has been reported in nine (9%) of the 99 cases reported from 1959 to 2018. Previous estimates suggest that pseudoaneurysm formation has an incidence of 14% [9], is theorized to develop within two to three weeks after the traumatic event [20], and has been identified on repeat angiographic imaging between four weeks to three months [12,20]. Because pseudoaneurysm carries a substantial risk for morbidity [2], aggressive angiographic surveillance and management should always be a facet of care for these types of PHI.

## Conclusions

This remarkable case highlights the truly unique nature of a single nail-gun-related PHI with a surprisingly benign clinical course. The literature is rich with descriptions of variations in nail location, complications, and management techniques. Although the nail head can often be accessed in many instances without requiring an intracranial exposure, this procedure should be considered for those nail-gun-related PHI cases that involve the skull base, with the nail projecting into the intracranial compartment, given the risks associated with trauma to the cerebral vasculature or the need for hemostasis upon removal. It is important to be aware of the possibility of immediate or delayed development of a pseudoaneurysm, necessitating preoperative and follow-up angiography.
